# Presence of Cytomegalovirus Infection Is Associated With an Unfavorable Outcome in Immunocompetent Infants With Pertussis

**DOI:** 10.3389/fcimb.2022.800452

**Published:** 2022-02-18

**Authors:** Wujun Jiang, Sainan Chen, Lina Xu, Xueyun Xu, Li Huang, Yuqing Wang, Chuangli Hao

**Affiliations:** Children’s Hospital of Soochow University, Suzhou, China

**Keywords:** human cytomegalovirus, pertussis, infants, plasma, BAL

## Abstract

**Background:**

We aimed to examine cytomegalovirus (CMV) infection in immunocompetent infants with pertussis, based on polymerase chain reaction in plasma and broncho alveolar lavage (BAL), and to assess the clinical characteristics and outcomes for these patients.

**Methods:**

We performed a prospective observational cohort study of consecutive infants with pertussis in Children’s Hospital of Soochow University between Jan 2017 and Jan 2020. We report the burden of CMV PCR in plasma and BAL within this patient group, and evaluate associations between CMV infection and pertussis in these hospitalized infants.

**Results:**

During the study period, 1,867 infants <1 years were evaluated for pertussis, 190 infants were diagnosed as pertussis. For the 190 pertussis patients, 38 (20.0%) patients had positive CMV PCR in plasma. CMV PCR in plasma had high sensitivity and specificity for CMV PCR in BAL (81.3% and 94.4%, respectively). Children with positive CMV PCR in plasma were 3.67 times more likely to present with severe disease (OR 3.67; CI 1.61-8.36). Comparisons of duration of hospital stay curves using the log-rank test statistic demonstrated that the relative risk of longer hospital stay of positive CMV PCR relative to negative CMV was 1.51 (95% CI: 1.05 to 2.14, P = 0.01).

**Conclusions:**

Our study reported a high prevalence of CMV reactivation in immunocompetent infants with lower respiratory tract infection. The presence of CMV in plasma may be associated with an unfavorable outcome in infants with pertussis.

## Background

Pertussis represents a serious and lethal threat to infants (Kilgore et al., 2016). Previous study reported that infantile pertussis infection had higher risk of hospitalization and mortality compared to the other age groups (Mbayei et al., 2019). Infants hospitalized for pertussis usually present with persistent cough, apnea and pneumonia.

Human cytomegalovirus (CMV) infections are also common in children, especially in infants (Bate et al., 2010). Our previous study found that CMV in broncho alveolar lavage (BAL) was detected in 51.4% of patients admitted for recurrent wheezing (Sun et al., 2018). Jeena et al. found that CMV was positive in 67% of the children with pneumonia admitted for mechanical ventilation (Jeena et al., 2017). In immunocompetent children, CMV usually underwent latent infections and subclinical replication. During latent infections, CMV produces immunosuppressive cytokines, inhibits T cell cytotoxic functions and has the ability to remain dormant in T cells in the lung. Evidence continues to accumulate that CMV infection could alter the host defenses and increase susceptibility to other infections, especially respiratory infections. Previous studies also reported that the presence of CMV infection were associated with slow-resolving respiratory infections in infants (Wejse et al., 2001; Cinel et al., 2014).

The clinical presentation of active CMV lung infections in infants included persistent cough, apnea and hypoxemia (Restrepo-Gualteros et al., 2019). These symptoms overlap with pertussis in infants. Thus, the associations between infantile CMV lung infection and pertussis are of great interest. We assumed that CMV infections may play a role in patients with pertussis.

In this study, we aimed to examine CMV infection in immunocompetent infants with pertussis, based on polymerase chain reaction (PCR) in plasma, and to assess the clinical characteristics, and outcomes for these patients.

## Method

### Study Population

We performed a prospective observational cohort study of consecutive infants with pertussis in Children’s Hospital of Soochow University between Jan 2017 and Jan 2020. Infants with the following conditions were excluded: history of congenital CMV infection, congenital heart disease with abnormal hemodynamics, inborn metabolic diseases, immunocompromised diseases, and extreme leukocytosis that need exchange transfusion or extracorporeal membrane oxygenation. The institutional review board of Children’s Hospital of Soochow University approved this study.

### Data Collections

The following parameters were recorded: demographics, clinical symptoms, laboratory parameters, and length of hospital stay. Pertussis was diagnosed by nasopharyngeal aspirates for PCR assays (Tatti et al., 2011). Patients with severe pertussis were considered if they developed apnoea or hypoxemia (Barlow et al., 2014). Molecular detection of CMV in plasma was performed by real-time quantitative PCR (San-sure Biotech, China) and a LightCycler 480 system (Roche Applied Science, USA), according to the manufacturer’s instructions. The detection limit for the test was 500 copies/mL.

### BAL Evaluation for CMV

The decision of whether to perform bronchoscopy with BAL was made by the attending pediatricians. A flexible bronchoscope (Olympus CV260, Tokyo, Japan) was used to assess primary abnormalities of the airways and clear all secretions or mucus plugs. In brief, the BAL was performed in the right middle lobe when diffuse infiltrates were evident on radiography; otherwise, it was performed at the site of localized infiltration. The recovered BAL fluid was pooled; 60%-80% of the instilled volume was recovered. BAL was collected by a sterile sputum-collecting pipe (Falcon 50 ml, Becton-Dickinson, Rutherford, NJ, USA) for microbiological analysis.

### Ethical Considerations

The study was approved by the Ethics Committee of Children’s Hospital of Soochow University (No: 2016026). It was conducted in accordance with Good Clinical Practice guidelines. Legal guardians of the study participants were counselled on the study prior to obtaining a signed informed consent.

### Statistics

Descriptive statistics were used to summarize results. Nonparametric data were reported as medians and interquartile ranges. Non-normally distributed continuous variables between groups were compared using Mann-Whitney U-test. Frequency distributions were compared using the Chi-squared test. We assessed our primary exposure (plasma CMV status), outcomes (severe disease and longer hospital stay), and potential confounders (including age, sex, birth weight, vaccination status and white blood cell [WBC] count) using univariate and bivariate statistics. To assess positive plasma CMV with respect to outcome, variables that were significant at the P <.1 level in unadjusted analyses were put into multiple logistic regressions. Predictor variables significant at P <.05 were retained in the final model, adjusted odds ratios were calculated, and model fit was assessed *via* the Hosmer-Lemeshow test. Duration of hospital stay was analyzed using the Kaplan-Meier method and the log-rank test for univariate comparisons among defined subgroups of patients based on clinical and laboratory characteristics. Multivariate analysis of hospital stay duration was performed using Cox’s proportional hazards regression mode. Data were analyzed by SPSS 22.0 (IBM, Armonk, NY).

## Results

### Study Population

**Figure 1:**
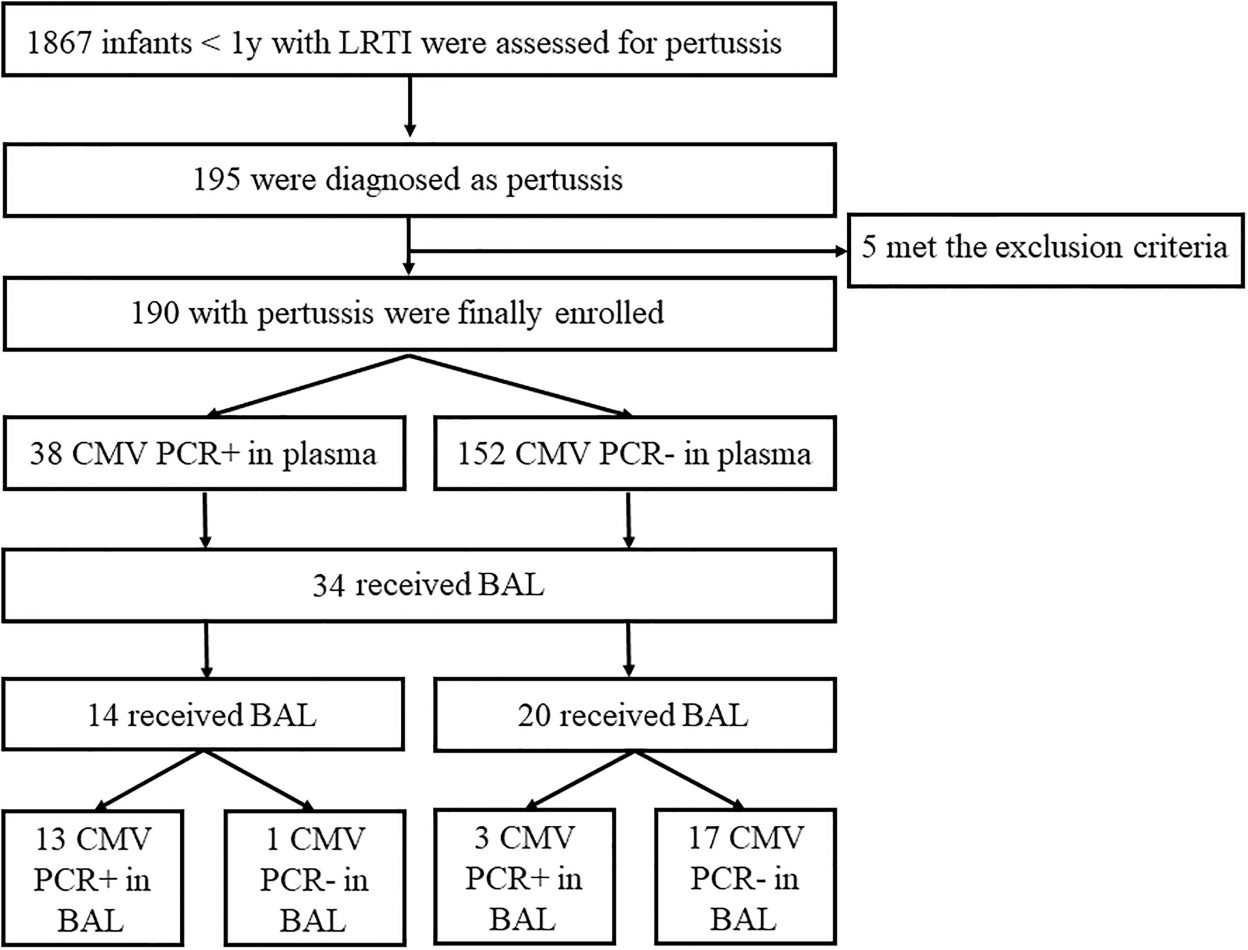
Study enrollment and Final Pertussis and Cytomegalovirus (CMV) Cases. LRTI, low respiratory tract infection.

During the study period, 1867 infants <1 years were evaluated for pertussis, 190 infants were diagnosed as pertussis (**Figure 1**). Of the 190 infants, 105 (55.3%) were males and 85 (44.7%) were females. The male to female ratio was 1.2:1. The median age was 3 months (range from 2 to 5 months). Positive CMV PCR in plasma was detected in 38 (20.0%) patients. The male to female ratio was 1.8:1. The median age was 2 months (range from 2 to 3 months). The virus load ranged from 5×10^2^ to 1.51×10^4^ copies/ml with a median of 1.6×10^3^copies/ml (**Table 1**). Comparison with these routine admissions data showed that there were no differences between the study children and admitted population in terms of age, sex, positive CMV rate and CMV virus load (all P>0.05, **Table 1**).

**Table 1:** Comparison of key descriptive variables between pertussis PCR-positive and negative cases.

Variable	Pertussis PCR-Positive Cases [n(%)=190]	Pertussis PCR-Negative Cases [n(%)=1672]
Males	105 (55.3)	1024 (61.2)
Median age (IQR), mo	3 (2-5)	3 (2-4)
Positive CMV in plasma	38 (20.3)	368 (22.0)
CMV load in plasma, copies/ml	1.6×10^3^ (5×10^2^ to 1.51×10^4^)	1.9×10^3^ (8×10^2^ to 6.2×10^3^)

### Comparison of Clinical Characteristics and Laboratory Values by CMV Status in Plasma

Pertussis cases with CMV viremia were younger than those without CMV viremia (2 [2-3] vs 3 [2-5], P=0.02). They presented a longer cough duration before admissions (10 [9-15] vs12 [10-15]). There were no differences in terms of sex, birth weight and vaccination status between infants with and without CMV viremia (all P>0.05). Patients with CMV viremia possessed a higher percentage of CD3+CD8+ T cells (26% vs 22%, p = 0.01) as well as a lower percentage of CD4+/CD8+ ratio (1.4 vs 2.1, p <0.01, **Table 2**).

**Table 2:** Clinical characteristics, laboratory values and their associated cytomegalovirus (CMV) PCR results in plasma.

Variable^†^	Negative CMV PCR (n = 152)	Positive CMV PCR (n = 38)
Males	83 (54.6)	22 (57.9)
Age, mo*	3 (2-5)	2 (2-3)
Birth weight, kg	3.3 (3.0-3.6)	3.4 (3.0-3.8)
*B. pertussis* vaccination	71 (46.7)	16 (42.1)
Cough duration before admission, d	10 (9-15)	12 (10-15)
White blood cell counts, ×10^9^/L	20 (14-28)	19 (15-28)
Lymphocytes%	62 (52-72)	71 (54-77)
Elevated Alanine aminotransferase, %	4 (2.6)	3 (7.9)
Albumin, g/L	43 (41-46)	43 (40-46)
Subpopulation of lymphocytes		
CD3^+^, %	69 (63-73)	66 (59-71)
CD3^+^CD4^+^, %	38 (33-40)	40 (36-47)
CD3^+^CD8^+^, %*	26 (21-32)	22 (15-24)
CD4^+^/CD8^+^*	1.4 (1.1-2.1)	2.1 (1.7-2.7)
CD19^+^CD23^+^, %	9 (6-15)	13 (7-15)
CD3^-^CD19^+^, %	22 (16-31)	26 (22-34)
CD3^-^CD16^+^CD 56^+^, %	7 (4-12)	6 (3-7)
Coinfection with respiratory viruses	50 (32.9)	15 (39.5)

^†^Data are presented as No. (%) or median (IQR).

*Significant differences (P <.05) were observed between the two groups.

### Comparison of CMV PCR Results in BAL by Plasma CMV Status

**Figure 2:**
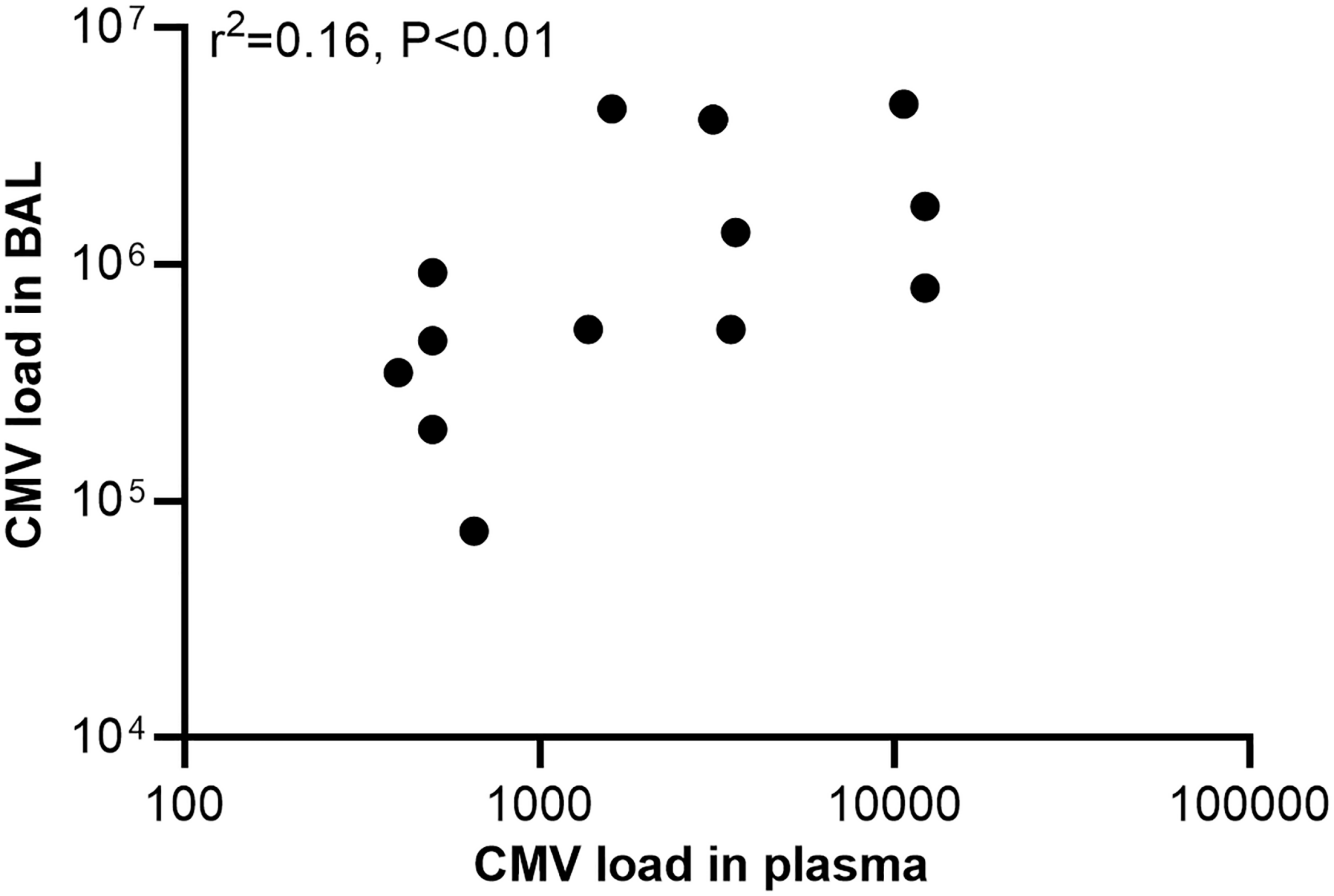
Regression plot comparing concordant detection of cytomegalovirus (CMV) DNA in plasma and bronchoalveolar lavage (BAL) samples. CMV load was recorded as CMV DNA copies per milliliter.

BAL was performed in 34 (17.9%) cases with pertussis (14 with positive CMV PCR and 20 with negative CMV PCR in plasma, **Figure 1**). The median age was 3 months (IQR, 2-5); 52.9% were male. Detection of CMV was positive in 16 (47.1%) out of 34 BAL samples with a median value of 5×10^5^ (IQR 6×10^4^–2×10^6^) copies/mL. Analysis of the 34 paired samples (BAL and plasma samples) was then performed. For infants with positive CMV PCR in plasma (n=14), 13 (92.9%) had CMV detected in BAL, while for those with negative CMV in plasma (n=20), only 3 (15%) had CMV in BAL. CMV PCR in plasma had high sensitivity and specificity for CMV PCR in BAL (81.3% and 94.4%, respectively, **Table 3**). Analysis of paired positive samples (n=13) found a strong correlation between the levels of CMV DNA in plasma and BAL (Spearman r^2^ = 0.16; P<0.01; **Figure 2**).

**Table 3:** Cytomegalovirus (CMV) detection by polymerase chain reaction (PCR) analysis of bronchoalveolar lavage (BAL) and plasma samples.

	No. of BAL samples (n = 34)			
Plasma sample PCR results	CMV positive	CMV negative	Sensitivity, %	Specificity, %
CMV positive	13	1		
CMV negative	3	17	81.3	94.4%

### Treatment and Clinical Outcomes

All the infants received macrolides, 55(28.9%) received gamma globulin. 35 (18.4%) received oxygen therapy. For the 38 with positive CMV PCR in plasma, only 5 (13.2%) received the treatment of ganciclovir. Paroxysmal cough and hypoxemia disappeared during the treatment period. Radiological improvement can be seen after the ganciclovir therapy.

**Figure 3:**
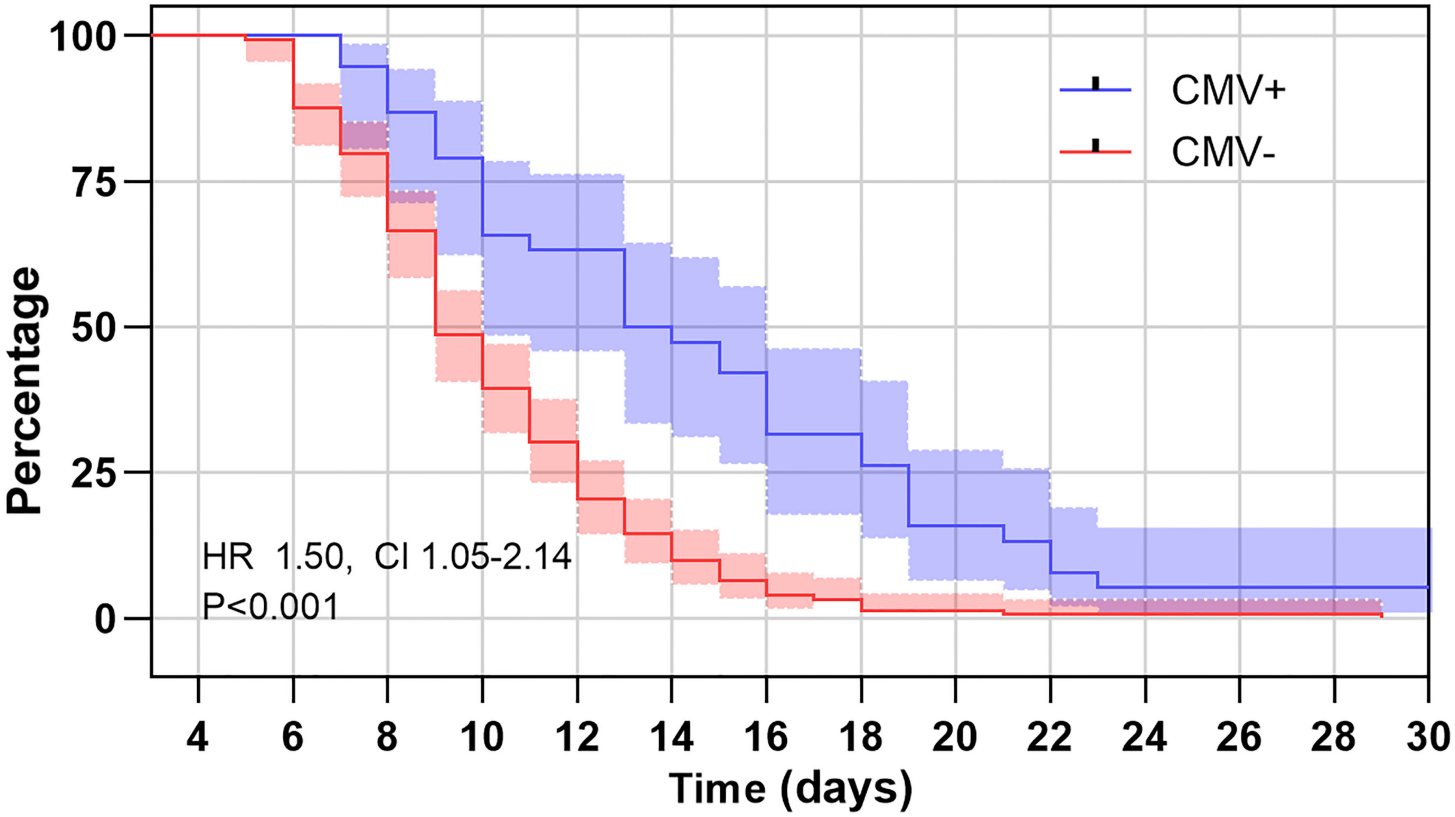
Kaplan–Meier estimates of length of hospital stay for B. pertussis patients with positive cytomegalovirus (CMV) PCR in plasma and those with negative CMV PCR. The log rank test for trend showed significant association between those with positive CMV PCR and negative CMV PCR (P < 0.01).

Of the 190 infants, 85 (44.7%) had length of hospitalization >10 days, 41 (21.6%) had length of hospitalization >14 days. After adjusting for age and vaccination status, children with positive CMV PCR in plasma were 2.80 times more likely to have length of hospitalization >10 days, and 5.89 times more likely to have length of hospitalization >14 days than those with negative CMV PCR results (OR 2.80; CI 1.32-5.95 and OR 5.89; CI 2.65-13.07, respectively, **Table 4**). Comparisons of duration of hospital stay curves using the log-rank test statistic demonstrated a statistically significant association between rate of regression and CMV status. After adjusting for age and vaccination status, the relative risk of longer hospital stay of positive CMV PCR relative to negative CMV was 1.51 (95% CI: 1.05 to 2.14, P = 0.01, **Figure 3**). While for the effect on disease severity, children with positive CMV PCR in plasma did not affect the clinical severity in those with B. pertussis-associated infection (OR 1.81; CI 0.85-3.86, **Table 4**).

**Table 4:** Associations of cytomegalovirus (CMV) PCR results in plasma with clinical outcomes, multiple logistic regression analysis.

Outcome	Positive CMV [n(%)=38]	Negative CMV [n(%)=152]	OR (95% CI)	Adjusted OR (95% CI)^a^
Severe disease				
Yes	11 (28.9)	34 (22.4)	1.81 (0.85-3.86)	1.82 (0.84-3.91)
No	27 (71.1)	118 (77.6)	Referent	–
Length of hospitalization >10d				
Yes	25 (65.8)	60 (39.5)	2.95 (1.40-6.21)	2.80 (1.32-5.95)
No	13 (34.2)	92 (60.5)	Referent	–
Length of hospitalization >14d				
Yes	19 (50.0)	22 (14.5)	5.91 (2.71-12.89)	5.89 (2.65-13.07)
No	19 (50.0)	130 (85.5)	Referent	–

a Multivariable analysis was adjusted for age and the vaccination status.

CI, confidence interval; OR, odds ratio.

## Discussion

Two important findings reported in our study are as follows: (1) we reported a high prevalence of CMV infection in immunocompetent infants with our study reported a high prevalence of CMV reactivation in immunocompetent infants with lower respiratory tract infection; (2) CMV infection were associated with negative outcomes in infants with pertussis.

Active CMV infections in premature infants, especially those born < 30 weeks gestational age and < 1500 g, are usually severe and may cause pneumonia and sepsis-like syndrome (Josephson et al., 2014; Kelly et al., 2015). However, in healthy term infants, active CMV infections are always asymptomatic or limited to mild non-specific illness (Coclite et al., 2013; Azenkot et al., 2019). Recent studies found that CMV might have pervasive negative impact on health through indirect effects on the immune system. In particular, CMV has increasingly been recognized as a potential cause of disease in immunologically normal adults with critical illness. While in children, there are very few data available about active CMV infection in immunocompetent children. In a prospective study that included 53 HIV-uninfected South African children ≤2 years old admitted to ICU with severe pneumonia, 12 (23%) had CMV DNA detection in BAL fluid (Govender et al., 2017). In the present study, we report the role of CMV infection in infants with pertussis for the first time. We found that 20% of the enrolled patients had CMV infection. The CMV infection rate was similar to the previous study.

Results of CMV in BAL and plasma samples were analyzed in our study. Interestingly, CMV PCR in plasma had high sensitivity and specificity for CMV PCR in BAL. The median CMV load in BAL was significantly higher than that in plasma. Analysis of paired positive samples found a strong correlation between the levels of CMV DNA in plasma and BAL. This result supported the concept of compartmentalization and the ‘spill-over’ effect of the pathogenic response. Having CMV disease (e.g., in lungs, liver or kidneys) should result in higher viral loads in each organ system, with a spill-over effect into the blood once the virus cannot be controlled in the target organ (Slyker et al., 2014; Tembo et al., 2015; Govender et al., 2017). Although CMV DNA in plasma and BAL were common in those patients, none of the patients in the present study had CMV retinitis, only 3 had elevated ALT. Therefore, we think that CMV caused illness that localized to the lower airways only and was not disseminated, as the patients included in the study were immunocompetent infants.

**Figure 4:**
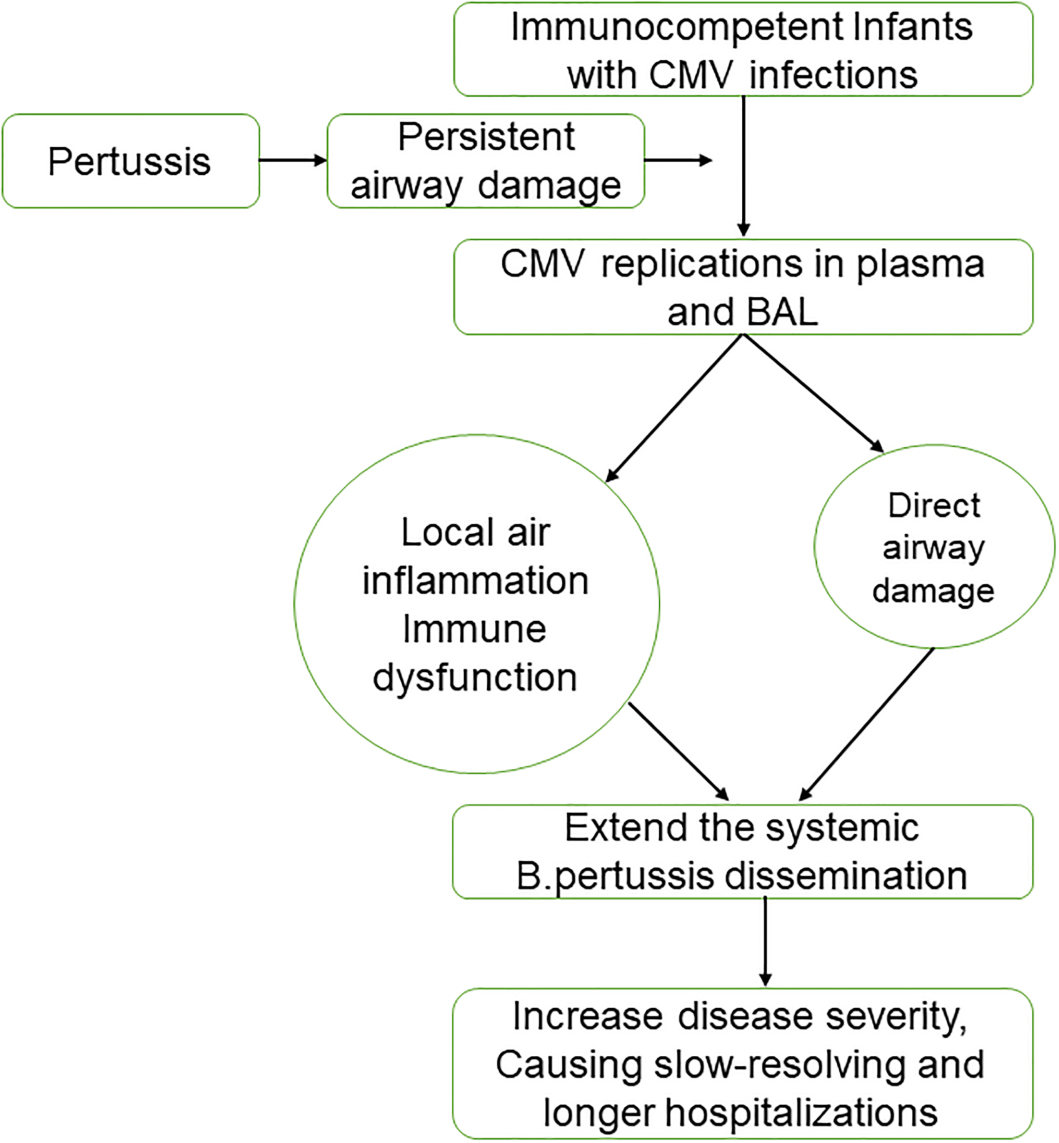
Hypothesized model for cytomegalovirus (CMV) reactivation in infants with pertussis. BAL, bronchoalveolar lavage.

In our study, CMV infection was associated with slow-resolving and longer hospitalizations in patients with pertussis. In studies about patients with compromised diseases, CMV load is associated with disease progression (Deayton et al., 2004; Griffiths et al., 2016). Cinel et al. reported that wheezing infants with CMV infections have a higher rate of severe respiratory diseases (Cinel et al., 2014). Jeena et al. found that HIV-infected children on mechanical ventilation with active lung CMV infection have poor outcomes (Jeena et al., 2017). The mechanism of this effect has not been fully understood, involving direct CMV pathogenicity or CMV-mediated lung injury (Vergara et al., 2018). The severity of disease depends on several factors, including the patient’s age, strength of the immune response, and extent of systemic bacterial dissemination (Kilgore et al., 2016). For infants detected CMV in BAL, CMV may affect the cell immunity, reduce the local immune responses and extend the systemic bacterial dissemination. As infants with pertussis usually have long disease duration, CMV reactivation could be initiated in these patients, and CMV replication then might in turn drive more inflammation in a feed-forward manner (Alyazidi et al., 2018). This will cause slow-resolving and longer hospitalizations (**Figure 4**).

There were limitations in our study. First, we could not get the BAL analysis in mild patients with pertussis who did not receive the bronchoscopy. Second, the number of patients enrolled was relatively small. Cohort studies enrolled larger number of patients are needed to understand the role of CMV in immunocompetent infants with pertussis. Third, our study did not study the molecular mechanism to address the difference between the CMV positive and CMV negative in immunocompetent infants with pertussis.

## Conclusions

Our study reported a high prevalence of CMV reactivation in immunocompetent infants with Our study reported a high prevalence of CMV reactivation in immunocompetent infants with lower respiratory tract infection. The presence of CMV in plasma may be associated with an unfavorable outcome in infants with pertussis.

## Data Availability Statement

The original contributions presented in the study are included in the article/supplementary material. Further inquiries can be directed to the corresponding authors.

## Ethics Statement

The studies involving human participants were reviewed and approved by the Ethics Committee of Children’s Hospital of Soochow University. Written informed consent to participate in this study was provided by the participants’ legal guardian/next of kin.

## Author Contributions

WJ and SC (co-author) developed the first draft and edited and wrote the first manuscript. YW, XX, LX, LH, and CH designed the study, analyzed data, and participated in writing the manuscript. All authors participated in data interpretation. YW revised and edited the final version of manuscript. All authors approved the final version of manuscript.

## Funding

This work was supported by a grant from the National Natural Science Foundation of China (Grant No.81573167, NO. 81971490), Social Development, Science and Technology Projects of Jiangsu Province (Grant BE2019671) and the Science and Technology Program of Suzhou (SYS2020069, SKY2021009).

## Conflict of Interest

The authors declare that the research was conducted in the absence of any commercial or financial relationships that could be construed as a potential conflict of interest.

## Publisher’s Note

All claims expressed in this article are solely those of the authors and do not necessarily represent those of their affiliated organizations, or those of the publisher, the editors and the reviewers. Any product that may be evaluated in this article, or claim that may be made by its manufacturer, is not guaranteed or endorsed by the publisher.
